# Sociodemographic characteristics predict land use patterns by farmers near a protected area in Madagascar

**DOI:** 10.1038/s41598-026-40592-6

**Published:** 2026-02-22

**Authors:** Kayla M. Kauffman, Michelle Pender, Jean Yves Rabezara, Prisca Rahary, Mark Janko, Lev Kolinski, Tyler Barrett, Maheriniaina Toky Randriamoria, James Moody, Voahangy Soarimalala, David López-Carr, Randall Kramer, Georgia Titcomb, Charles Nunn

**Affiliations:** 1 Department of Ecology, Evolution, and Marine Biology, University of California Santa Barbara, Santa Barbara, CA USA; 2 Department of Evolutionary Anthropology, Duke University, Durham, NC USA; 3 Duke Global Health Institute, Durham, NC USA; 4 Association Vahatra, BP 3972, 101 Antananarivo, Madagascar; 5 Mention Zoologie et Biodiversité Animale, Domaine Sciences et Technologie, Université d’Antananarivo, BP 906, 101 Antananarivo, Madagascar; 6 Department of Sociology, Duke University, Durham, NC 27708 USA; 7 Institut des Sciences et Techniques de l’Environnement, Université de Fianarantsoa, BP 1264, 301 Fianarantsoa, Madagascar; 8 Department of Geography, University of California Santa Barbara, Santa Barbara, CA USA; 9 Nicholas School of the Environment, Duke University, Durham, NC USA; 10 Department of Fish, Wildlife, and Conservation Biology, Colorado State University, Fort Collins, CO USA

**Keywords:** Land use, Movement ecology, Sociodemographics, Madagascar, Smallholder farmer, One health, Conservation biology, Ecological epidemiology, Environmental impact, Psychology and behaviour, Socioeconomic scenarios

## Abstract

Globally, most farms are smaller than 10 hectares. Land use by these smallholder farmers in agricultural frontiers is crucial to conservation, food security, and exposure to infectious diseases. However, solely survey-based land use studies miss the fine scale movements that collectively form an individual’s land use and thus environmental exposures, particularly in fragmented landscapes. We applied movement ecology-based approaches with GPS and survey data to investigate how sociodemographic variables corresponded with land use among farmers living adjacent to Marojejy National Park in northeastern Madagascar. Our data on 902 individuals spanning 3 years revealed striking differences in land use by gender and socioeconomic status. Men traversed 65% (95% CI [51%, 82%]) more area and spent 30% less time (95% CI [20, 41%]) in the village than women. Socioeconomic status, educational attainment, and having young children covaried with time spent in secondary forests where vanilla is grown, swidden areas where slash-and-burn farming methods are used, and the national park. By revealing the extent to which social and demographic variables predict individual movement patterns, our work identifies targets for addressing emerging diseases and health disparities. The GPS and analytical approaches developed here can be applied elsewhere to provide policy insights and to understand variation across human-ecological systems.

**Supplementary Information:**

The online version contains supplementary material available at 10.1038/s41598-026-40592-6.

## Introduction

Approximately 43% of the world’s population lives in rural areas, and smallholder farming is the primary occupation in these communities^1^. Globally, 80% of food is produced by family farms^1,2^. Rural communities of smallholder farmers, particularly in low and middle-income countries (LMICs), are disproportionately affected by climate change, food insecurity, and environmentally transmitted infectious diseases^3–6^. Land use and land cover change by frontier farmers falls at the nexus of emerging zoonotic disease risk as routine behaviors by individuals shift ecological assemblages, and time spent in these areas results in altered disease exposures. Thus, understanding how sociodemographic factors influence people’s movements across the landscape in rural contexts is crucial for guiding public health measures to prevent or reduce infectious disease transmission, to guide agricultural policies, and to obtain legislative support for climate adaptation.

Human-environmental interactions (e.g., land use^7^) shape people’s identities, occupations, and socioeconomic positions and reveal disparities in food security and infectious disease exposures. Land use, migration, and movement decisions are particularly pertinent to the health of farmers in LMICs, who interact closely with the land. Over time, individual land use patterns contribute to deforestation as farmers expand into more fertile land or increase their reliance on cash crops. On a daily scale land use patterns impact individual disease exposures and reflect farming livelihoods and sociodemographic characteristics. Demographers study land use with surveys, deidentified GPS data, and agent-based models. Survey-based movement ecology methods are well-suited to capture individual perceptions of behavior, such as gender-based differences in range size^8,9^, yet perceived or self-reported behaviors often do not align with routine, daily land use patterns. For example, individual-level studies of land use in rural LMIC settings found that questionnaire-based data only weakly correspond with fine-scale GPS-based daily movement patterns^7,10^. Unlike surveys, detailed GPS-based studies reveal true movement (i.e. trajectories) which form land use patterns. GPS-tracking data is widely used in urban settings to infer demographics for urban planning and marketing purposes^11^, reveal disparities of land use^12^, inform social and economic research^13^, and plan healthier cities^14^. Integrating GPS-based data on individual land use with remotely sensed land cover information can reveal exposures to environmentally transmitted infectious diseases. Thus, to develop a robust understanding of fine-scale predictors of human land use in rural LMIC contexts, it is necessary to combine survey and GPS data. This combined approach allows for the investigation of local disparities in wealth and exposures to infectious diseases, as well as long term land use and land cover change.

Movement ecologists investigate how animals interact and traverse their environments by studying their trajectories, internal and external drivers of movement, and mechanisms, such as navigation. Studies of animal movement in the “golden age of biologging” are fueled by the increased availability of GPS data-loggers, spatial data, and improved statistical modeling^15,16^. These movement ecology methodologies applied to human GPS data can be used to consider movement in relation to land use types, with the same limitation that time spent in an area is not necessarily synonymous with how important the area is to that individual. With that understanding, GPS-data are useful in developing quantitative land use metrics such as home range size and proportion of time spent in specific areas or land cover types, which can then be used to assess differences among demographic groups^17,18^. In the context of infectious disease, animal-animal and animal-environment contact networks have been used to infer disease exposure and transmission^19^. Other studies have also integrated landcover classifications with land use data to predict infection risk^20,21^. Furthermore, physiologically and behaviorally important resources can be found by identifying areas where animals spend time and aggregate^22,23^. These individual GPS-based approaches essential for capturing variation in how people interact with and shape their environment.

Here, we apply demographic and movement ecology methodologies to investigate the individual movement trajectories that form land use patterns among farmers living near the boundary of Marojejy National Park in northeastern Madagascar. Individuals in this area are predominately smallholder farmers (manage less than 10 ha^2^) and rely largely on family and daily wage labor to grow food for family consumption and cash crops for sale^24^. Land use types in the areas surrounding villages is primarily agrarian—vanilla, rice, and mixed crop farming—resulting in a highly fragmented, heterogeneous agroecosystem abutting the largely intact rainforest of the national park. People living in this region primarily rely on producing rice and vanilla alongside other crops for subsistence and income, and 76% of surveyed farmers reported challenges of food insecurity^4,25^. Households with fewer people and multiple income sources, including vanilla and surplus rice, are generally more food secure^26^.

Over 3 years, we conducted surveys and distributed GPS trackers to people in three villages to investigate how gender, age, and socioeconomic variables corresponded with land use. We hypothesized that women would have smaller home ranges than men because they have more responsibilities in the village, and that this difference would be greatest in households with young children. By collecting GPS tracker data alongside socio-demographic surveys, we also aimed to understand nuanced relationships between land use and other demographic traits, particularly wealth and income indicators. We expected that individuals who own and farm more land would have larger home ranges; however, if hired workers farm a person’s land, we expected the opposite trend. Individuals who grow a more diverse set of crops and farm in areas farther from their homes were expected to have larger home range sizes and to access more land cover types. Longer-term wealth indicators, such as house construction materials, purchased durable goods, and education (which represent intergenerational familial wealth), likely also influence land use. We expected wealthier individuals to spend more time in the secondary forest where vanilla, a labor-intensive and valuable cash crop, is grown. Trends observed for these long-term wealth indicators likely covary with shorter-term wealth indicators (income), such as growing cash crops and owning higher-value animals. With our movement ecology-based approach, we also explored if the proportion of an individual’s time in different land cover types is indicative of gender or wealth-based separations of labor.

## Results

### Survey

In total, 1,297 people participated in the study, representing 262 people from village A, 435 from village B, and 600 from village C. Of these, 902 people consented to wear a GPS logger for ≥ 1 week, resulting in 1 to 31 days of GPS data (mean ± sd = 6.2 ± 5.1, median = 5). Demographic information for all participants is provided in Table 1. Among GPS-wearing participants, 44.6% (402/902) were female, and significantly fewer females opted to wear a GPS in village B (p = 0.015). Almost all GPS-wearing participants (95.5%; 861/902) farmed one or more crops. Most individuals (88.0%, 769/874; 28 missing data) owned ≤ 10 ha, and only 14 individuals owned more than 30 ha (max = 120 ha). The average participant owned 6.5 ± 9 ha (median = 4 ha), however, estimates of landownership may have underestimated the amount of lower-quality land owned due to the phrasing of this survey question. School level among participants ranged from none to higher education, with significantly fewer people with no education opting to wear a GPS tracker in village B compared to those with secondary (p = 0.001) or higher (p = 0.012) education.

**Table 1:** Demographic traits of survey participants.

Trait	Village A			Village B			Village C		
	All	GPS	No GPS	All	GPS	No GPS	All	GPS	No GPS
N	262	182	80	435	328	107	600	392	208
Gender^B^									
Female	120; 0.72 (0.63,0.79)	86	34	182; 0.69 (0.62,0.76)	126	56	291; 0.65 (0.59,0.71)	190	101
Male	142; 0.68 (0.59,0.75)	96	46	253; 0.8 (0.74,0.84)	202	51	309; 0.65 (0.6,0.71)	202	107
Age	41.6 [18, 85] (1)	43.2 [18, 85] (1)	37.9 [18, 83] (0)	34.6 [18, 85] (1)	33.7 [18, 80] (1)	37.4 [18, 85] (0)	36.3 [18, 100] (0)	37 [18, 96] (0)	34.9 [18, 100] (0)
Household size	4.3 [1, 10] (1)	4.2 [1, 10] (1)	4.3 [1, 8] (0)	3.9 [1, 12] (0)	4.2 [1, 10] (1)	4.3 [1, 8] (0)	3.8 [1, 13] (0)	4.2 [1, 10] (1)	4.3 [1, 8] (0)
Main activity									
Farm crop	194; 0.69 (0.61,0.75)	133	61	264; 0.72 (0.66,0.78)	191	73	492; 0.65 (0.61,0.69)	321	171
Farm mixed	62; 0.71 (0.58,0.81)	44	18	124; 0.83 (0.75,0.89)	103	21	30; 0.8 (0.61,0.92)	24	6
Other	6	5	1	46; 0.74 (0.59,0.85)	34	12	76; 0.61 (0.49,0.71)	46	30
No response				1		1	2	1	1
Marital status									
Single	60; 0.75 (0.62,0.85)	45	15	47; 0.81 (0.66,0.9)	38	9 (1)	164; 0.65 (0.57,0.72)	107	57
Partner	202; 0.68 (0.61,0.74)	137	65	387; 0.75 (0.7,0.79)	290	97	428; 0.65 (0.6,0.7)	279	149
No response				1		1	8	6	2
Schooling									
None^B^	14; 0.86 (0.56,0.97)	12	2	21; 0.43 (0.23,0.66)	9 (1)	12 (2)	14; 0.36 (0.14,0.64)	5	9
Primary	158; 0.66 (0.58,0.73)	104	54	179; 0.72 (0.65,0.78)	129	50	243; 0.69 (0.62,0.74)	167	76
Secondary	67; 0.79 (0.67,0.88)	53	14	178; 0.81 (0.75,0.87)	145	33	254; 0.64 (0.58,0.7)	162	92
Higher	23; 0.57 (0.35,0.76)	13	10	54; 0.81 (0.68,0.9)	44	10	81; 0.64 (0.53,0.74)	52	29
No response				3	1	2	8	6	2
Land owned									
0 ha	0	6	0	16; 0.81 (0.54,0.95)	13	3	9	5	4
(0,10] ha	224; 0.69 (0.63,0.75)	155	69	360; 0.76 (0.71,0.8)	274	86	488; 0.65 (0.6,0.69)	316	172
> 10 ha	21; 0.76 (0.52,0.91)	16	5	43; 0.74 (0.59,0.86)	32	11	76; 0.75 (0.64,0.84)	57	19
No response	11	5	6	16	9	7	27	14	13

Columns indicate the community in which a participant lived, the summary of all survey participants, and split out by those that wore a GPS tracker and those that did not wear a GPS tracker. For categorical traits, the “All” column contains the number of individuals and the proportion that wore a GPS (95% confidence interval). The GPS and No GPS columns contain the number of individuals and. For the continuous traits (age and household size) the mean, [range] and (number of NA) are reported. ^ABC^Indicates villages with a significant difference (α = 0.05) between the participants that wore a GPS tracker and those that did not using a χ^2^ test with a Holm adjustment for categorical traits for all traits with ≥ 10 total individuals and an ANOVA for continuous traits.

Principal component analyses (PCA) of wealth revealed that wealth based on house construction materials and durable goods varied primarily along one principal component (PC). PC1 from the house materials PCA explained 70.6% of the variance and was primarily informed by the materials used to construct the floor and walls of the home. Having more valuable house construction materials was positively associated with the house materials PC1 (Pearson’s correlation = 0.96). The PC1 from the durable goods PCA explained 38.6% of the variance and was primarily due to owning a television, cell phone, using phone banking, and owning a radio. The durable goods PC1 was positively associated with the number of goods owned (0.97).

Both the crops grown and the animals owned PCAs split along two PCs describing their absolute number (PC1) and their value (PC2). The animals owned PC1 explained 30.4% of the variance and was correlated with the number of types of animals owned (0.77) and the total number of animals owned (0.77). The animals owned PCA also split along the PC2 axis, with negative values corresponding to cows (e.g. the high-value animal) and positive values with all other animals. The PC1 of the crops grown PCA explained 16.9% of the variance and had a strong positive correlation (0.84) with the total number of crops farmed. Again, the crops PCA split along the PC2 axis, with positive values corresponding to subsistence crops and negative values with cash crops and rice. The biplots for each PCA with convex hulls for each village are shown in Extended Data Fig. 1.

**Fig. 1:**
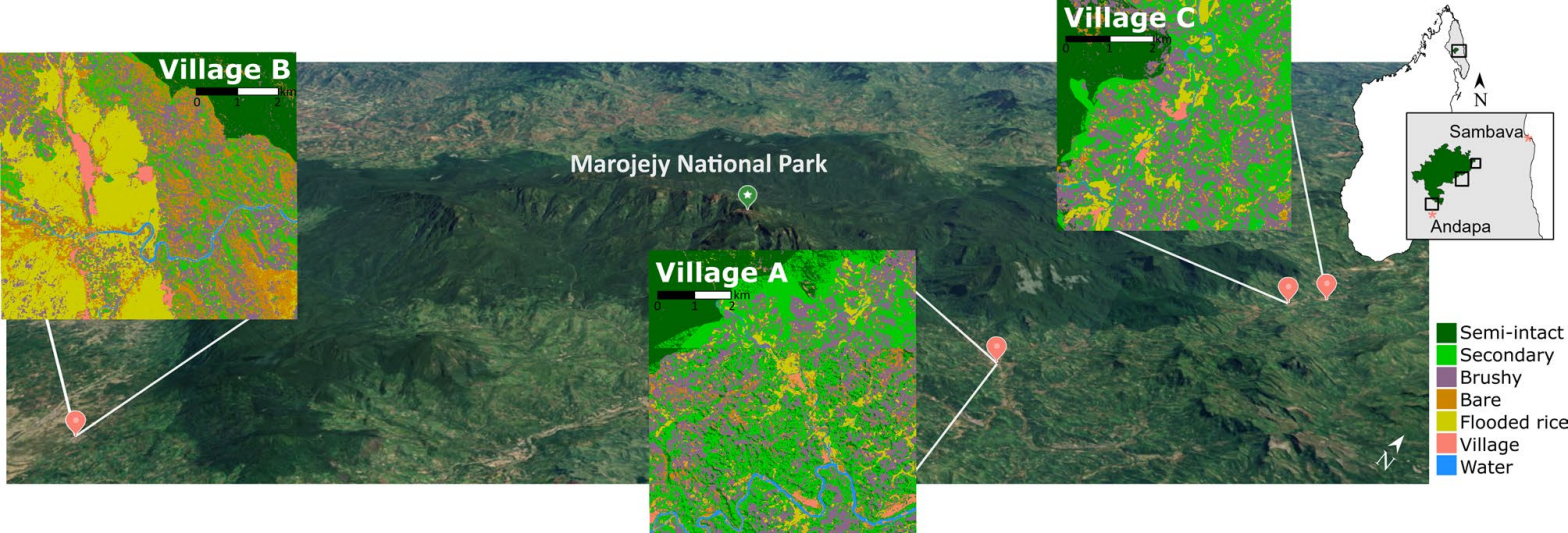
Google Earth image of the study area with insets of the land classifications in the areas immediately surrounding each village included in this study. The inset in the top right corner depicts the SAVA region (grey) of Madagascar (white), the green area is Marojejy National Park, and the black box shows the study areas for each village shown in the main figure. The shapefile of Madagascar was obtained from https://gadm.org and the shapefile for Marojejy National Park was obtained from OpenGIS. The Google Earth image was produced using Google SNES/Airbus Maxar Technologies Data SIO, NOAA, U.S. Navy, NGA, GEBCO.

### Land cover classification

A mosaic of cloud-free Sentinel II images captured between February 2020 and August 2021 were classified as semi-intact forest, secondary forest, brushy regrowth, bare ground, flooded rice, bare ground, and village following the methods used by Kauffman et al.^27^. Classifications were then manually edited in areas people used to ensure their accuracy. Specifically, bare areas inside the village were reclassified as village and all tree cover inside the national park was classified as semi-intact forest and outside of the park was classified as secondary forest.

Study area size varied among villages (A = 49.5 km^2^, B = 34.8 km^2^, C = 20.1 km^2^). The proportion of each land cover type also varied (Fig. 1, Supplementary Fig. 2, Supplementary Table 1). Notably, village A had more secondary forest (46.5%) compared to villages B (13.3%) and C (36.4%). Village B had more flooded rice (31.4%) than villages A (6.75%) and C (8.89%). Thus, individuals in these communities varied in their access to different cover types; however, the relative proportion of time spent in a land cover type did not correspond to land class availability (Extended Data Fig. 2). Land use and activities occurring in each land cover type are summarized in Table 2.

**Fig. 2:**
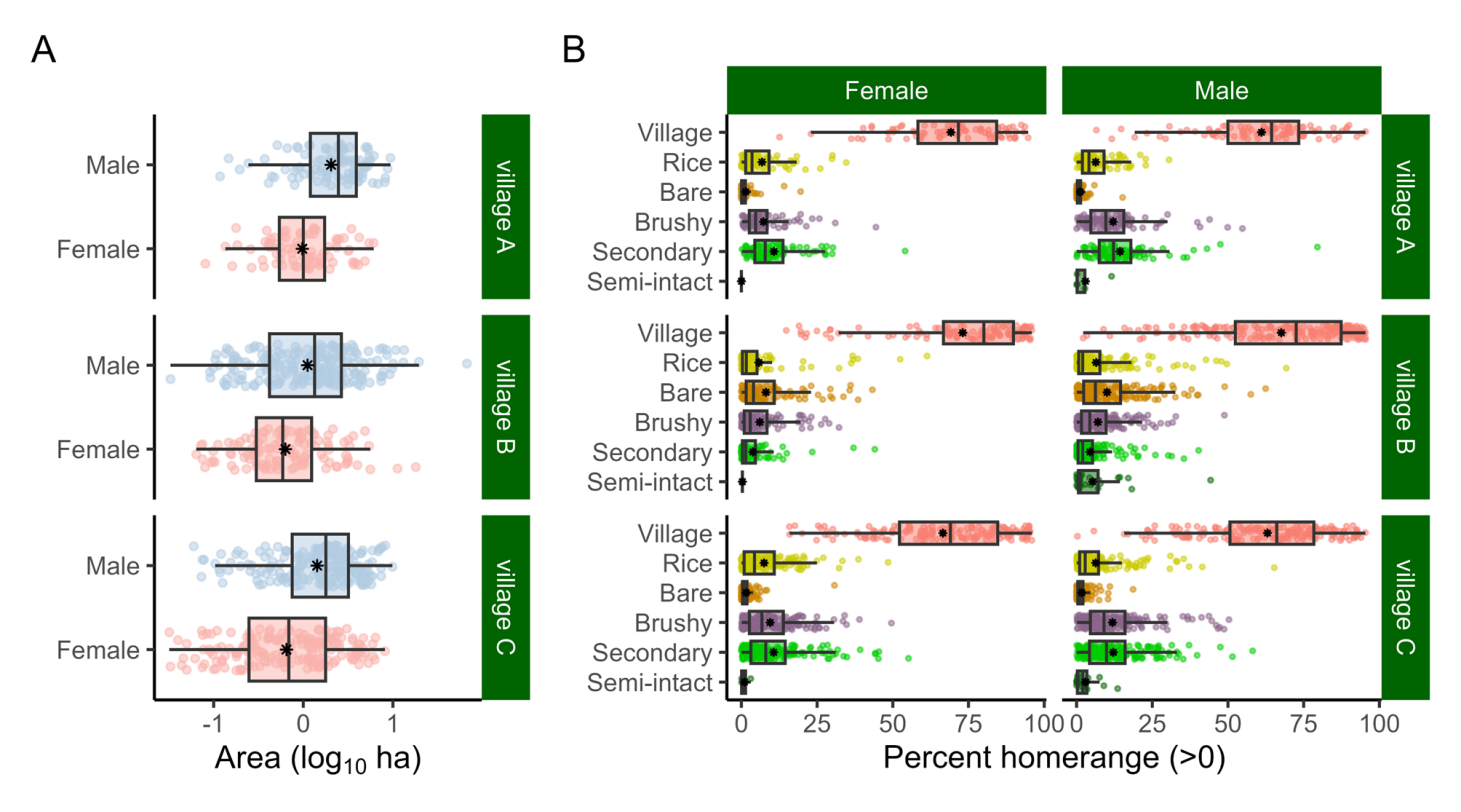
Boxplots of the (**A**) home range size and (**B**) time spent (percent of their daytime home range) in each land cover classification type by gender and study village. Fine-scale estimates of size and use were made possible by using GPS data loggers. Individuals who did not spend time in a cover type were removed from this plot for visual clarity. The “⁕” indicates the average.

**Table 2:** Land cover classifications based on remote image classifications and corresponding descriptions of land use types and activities in each class based on our observations and local knowledge.

Land cover classification	Description	Activities
Semi-intact forest	Tree cover within the boundary of Marojejy National Park	Logging, hunting, mineral collection
Secondary	Tree cover outside of Marojejy National Park. Predominately forest fragments, introduced fruit trees, and agroforest	Shade-grown vanilla, coffee, pepper, cloves, tree fruit, firewood collection
Flooded rice	Basins and flat, low-lying areas that are flooded most of the year	Rice
Village	Area where homes and other buildings are aggregated	Dwellings, sports field, small markets, small vegetable gardens
Brushy regrowth	Vegetation other than trees, including shrubs, grasses, lianas, and crops	Fallow fields, firewood collection, vegetables, and other crops (sugarcane, banana, non-shade grown vanilla, hillside rice)
Bare	Areas with minimal cover	Roads, vacant crop fields
Water	Permanent water sources (ponds, streams, rivers). All but the largest water sources are covered by trees (forest classifications)	Bathing, washing clothes and dishes, and some fish farming

### Home range size

Individual home range sizes varied by village and gender (Fig. 2A).

**Fig. 3:**
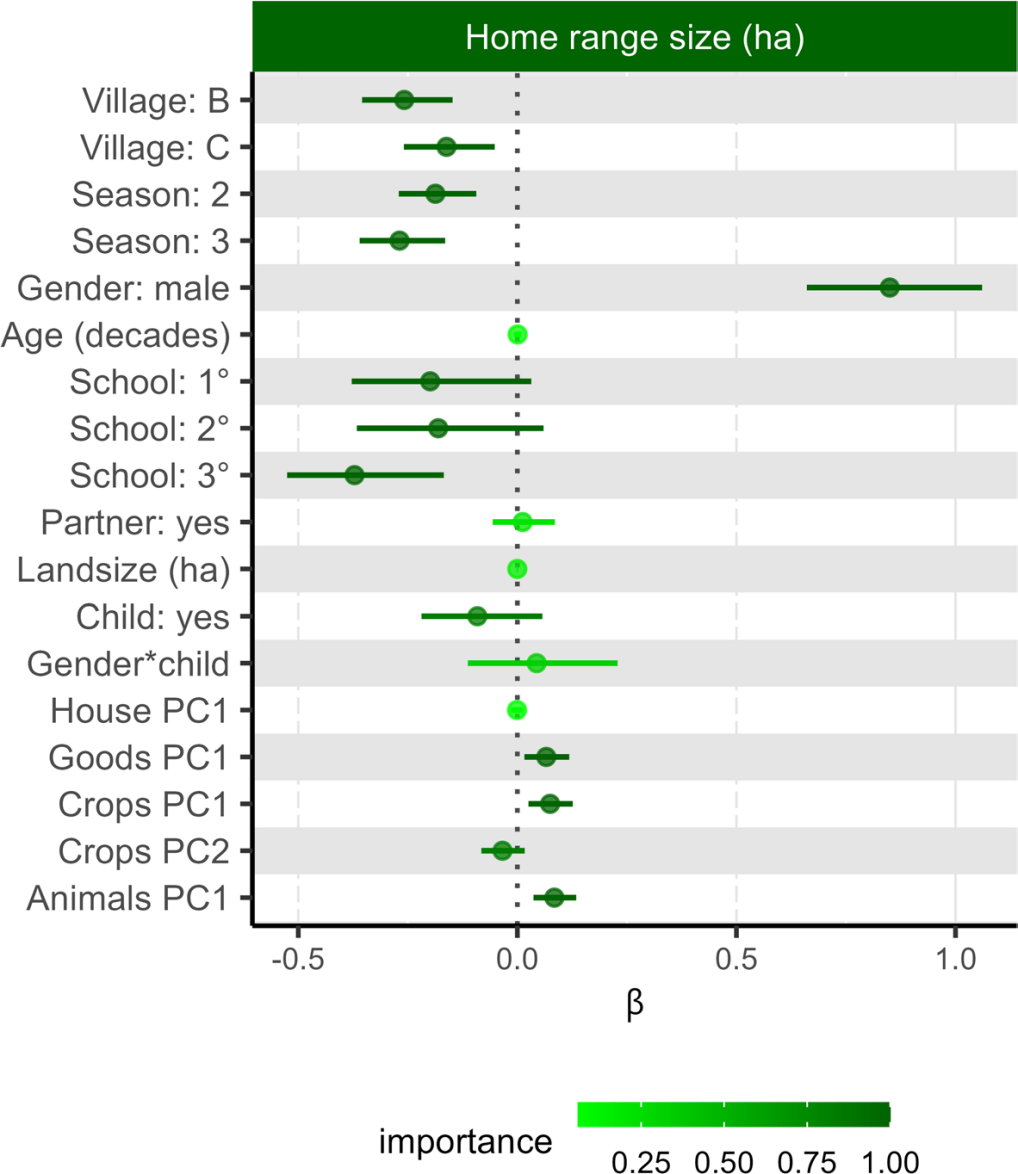
Home range size model coefficients with 95% confidence intervals based on the full model of the “best models subset” by ∆AICc ≤ 2. Β represents the association between each predictor and home range size in hectares. The predictor “Child” means the participant had a child ≤ 3 years old in their household. The shading represents the importance of each predictor and is based on the sum weight of each model containing the predictor in the best model subset (also shown in Supplementary Table 2).

The most important predictors of home range size, as determined by sum weight in the best models subset (∆AICc ≤ 2^28^), were village, season, gender, schooling, material wealth (goods PC1), and number of crops (crops PC1) and animals (animals PC1; Fig. 3, Supplementary Table 2). Having young children and crops PC2 were also important predictors, though they were not present in all models in the best model subset. Household size and animals PC2 were not included in the best model subset.

In the full averaged model, after accounting for seasonal and village differences we found that, as we predicted, men had larger home range sizes than women by 0.85 hectares (95% CI [0.66,1.06]; Fig. 3, Supplementary Table 3). Participants with more than secondary education had smaller home ranges than those with no education (estimated marginal mean ± SE: − 0.22 ± 0.06), a primary-level education (− 0.12 ± 0.03), and a secondary-level education (− 0.13 ± 0.03). Individuals, particularly women, from households with children also had smaller home ranges. Age and marital status did not strongly influence home range size and the wealth metric effects were mixed. Total land owned and house construction materials PC1 were unrelated to home range size. However, individuals with greater material wealth (goods PC1), more animals farmed (animals PC1), and more types of crops grown (crops PC1), particularly cash crops (crops PC2), had larger home ranges (Fig. 3, Supplementary Table 3).

We also observed differences in home range size among villages and seasons in the linear model. Post-hoc tests revealed significant differences between all three villages, with participants from village A having relatively larger home ranges compared to both B (difference = 0.13 ± 0.03 hectares) and C (0.06 ± 0.03 hectares) and participants from village C having slightly larger home ranges than those from village B (0.07 ± 0.02 hectares). This difference in home range size by village corresponds to village A having a larger study area than villages C and B; however, village B had a larger study area than village C. The season in which a participant was enrolled also impacted home range size, with being enrolled in the hot, dry season resulting in a 0.09 ± 0.02 hectare larger area compared to the warm, wet season and a 0.14 ± 0.03 hectare larger area compared to the transitional season. There was not a significant difference in home range size between the warm and wet season and the transitional season (0.06 ± 0.03 hectares).

### Time spent by land classification

Time spent in each land classification (proportion of daytime 95% utilization distribution^29^) varied greatly among land cover types, genders, and villages (Fig. 2B). People spent most of their time (66.5 ± 21.1%) in the village, and most individuals (93.3%, n = 802) did not enter the semi-intact forest.

**Fig. 4:**
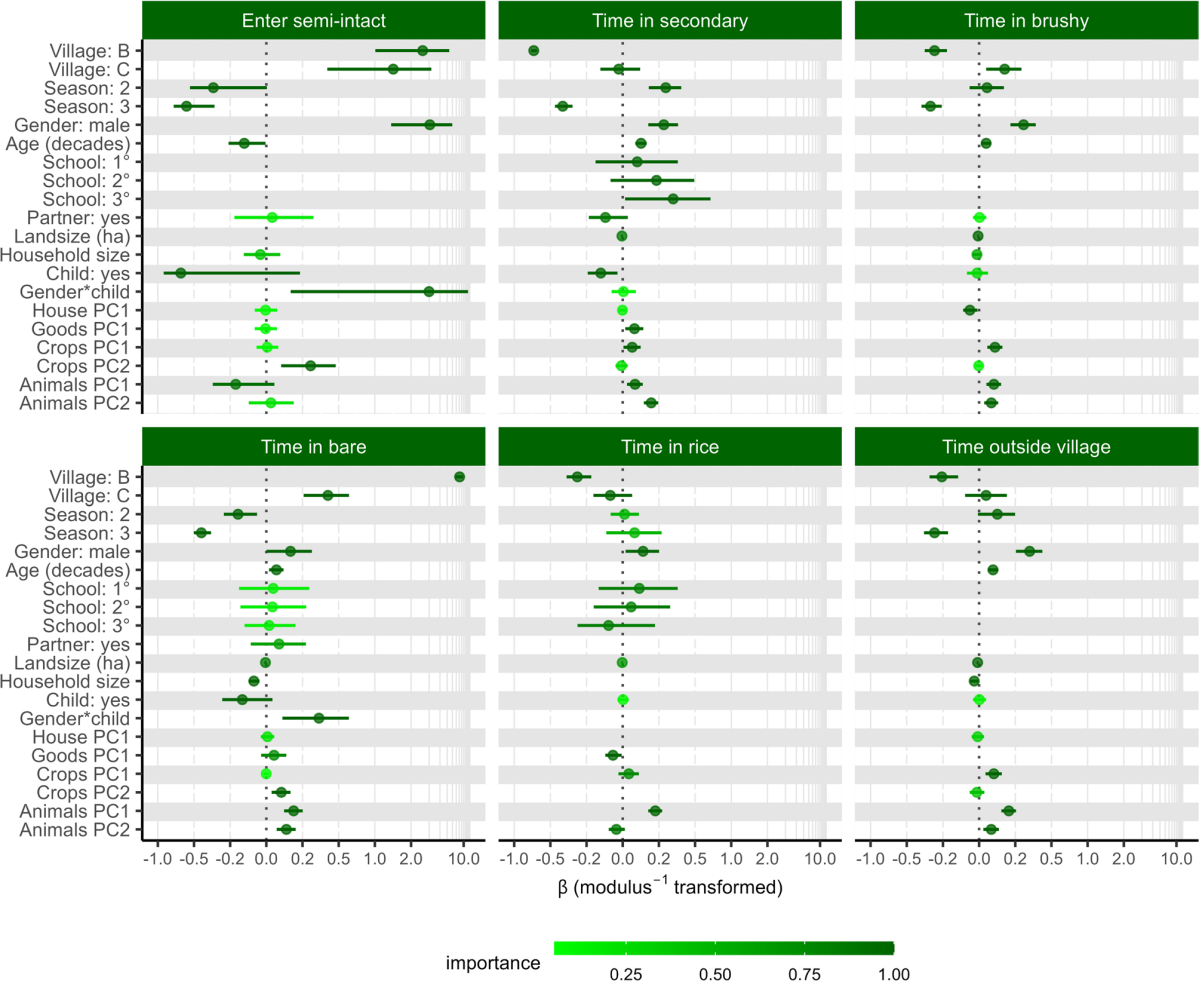
Coefficient plots of full model averaged effects on the proportion of time people spent in each land cover type or their change in odds of entering the semi-intact forest. The “best models subset” contained all models with ∆AICc ≤ 2. The predictor “Child” means the participant had a child ≤ 3 years old in their household. The shading represents the importance of each predictor and is based on the sum weight of each model containing the predictor in the best model subset (also shown in Supplementary Table 2). For visual clarity, the x-axis was modulus transformed (λ =  − 1).

Models of the proportion of individuals’ home range in each landcover type corresponded to landcover type availability in each village and provided insights into how demographic characteristics may impact land use patterns (Fig. 4, Supplementary Table 3). As hypothesized, participants’ gender remained important in all models, with men more likely to spend time in all landcover types outside of the village than women (Fig. 4, Supplementary Tables 2, 3). Age (in decades) was important in all land use type models except rice. Counter to our predictions, time spent outside the village increased with age, except in the semi-intact forest where the odds of accessing the forest decreased. Individuals with young children spent more time in the secondary forest, and men with children under 3 spent more time in bare areas. Individuals with more than a secondary-level education spent relatively more time in the secondary forest than all other levels (estimated marginal mean ± SE: secondary = 1.09 ± 0.07; primary = 1.22 ± 0.09; none = 1.32 ± 0.17). Other demographic traits had minimal correspondence with the time spent in the various land cover types.

People with more land and a greater variety of crops (PC1) and animals (PC1), regardless of type, spent more time outside the village, while lower value crops and animals (PC2s) primarily increased the odds that a person entered the semi-intact forest or spent time in brushy areas (Fig. 4, Supplementary Tables 2, 3). In contrast, long-term wealth indicators had small effect sizes; individuals with more land and material wealth spent less time in brushy regrowth and flooded rice fields and more time outside the village and in secondary forests.

The village and season in which the study took place were important (sw = 1) in all models, except for season in the model of time spent in flooded rice fields (sw = 0.48; Fig. 3). Some of the differences between the villages corresponded to the cover availability. For example, in village B there was substantially more bare ground and the odds of participants from village B spending time in bare ground compared to village A and village C was substantially higher (OR: 9.40 ± 0.72 and 6.73 ± 0.38, respectively). However, the time spent in flooded rice fields did not correspond to the relative amount of rice in the area surrounding each village; participants from village B, which had substantially more rice (Supplementary Fig. 2) spent less time in rice fields than those from village A and C (OR: 0.75 ± 0.04 and 0.79 ± 0.04 respectively). Cumulatively, the differences in time in different land classes resulted in participants from village B spending less time outside of the village compared to village A (OR: 0.80 ± 0.05), who in turn spent less time outside of the village than those from village C (OR: 0.77 ± 0.04). The effect of season likely corresponds to the harvest and planting seasons for some of the crops grown. In particular, the vanilla harvest occurs July through September^26^, and before harvest the crop is commonly guarded. This corresponds to participants spending more time in the secondary forest during our season 2 than the other seasons (OR season 1: 1.26 ± 0.06 and 3: 2.02 ± 0.12). After harvest vanilla is cured near people’s homes in the village, typically between July and September, the period in which (our season 3) we found participants spent less time in secondary forest (OR season 1: 0.62 ± 0.04 and 2: 0.49 ± 0.03) and overall, less time outside of the village (OR season 1: 0.75 ± 0.04 and 2: 0.69 ± 0.04).

## Discussion

Drawing on methods from movement ecology, we provide insights into how sociodemographic characteristics influence the home range size and time spent in different land cover types by Malagasy farmers. Our findings have broad implications for understanding how socioeconomic status, family composition, and demographic traits drive land use and land cover change in agricultural frontiers resulting in differential environmental exposures among individuals in vulnerable populations. We move beyond the well-described pattern that men have larger home ranges than women (e.g. Wood et al.^9^) to identify additional factors that influence individual variation in land use. Individuals who ranged over a greater area had lower educational attainment, owned more animals, farmed different crops, and had more material wealth. Similar sociodemographic traits were associated with the time that individuals spent in specific land use types—people with fewer agricultural and material resources spent more time in places where slash-and-burn (swidden) agriculture is used. These more nuanced insights into small differences in movement-based land use among rural farmers were possible due to using GPS data loggers, which provide more reliable data on land use than surveys, and allow for quantitative, high resolution measures of time and area, which is particularly necessary in highly fragmented land cover mosaics. The use of GPS data loggers also avoids errors or biases associated with recall, sampling, conformity, and data entry, thus enabling more accurate and quantitative estimations of home ranges.

Home range size and time spent in different land cover types were influenced by demographic traits rather than solely by the availability of land cover. Time spent in the village and agricultural areas varied by household characteristics, education level, and land owned. Interestingly, the differences in time spent in particular cover types between genders were smallest in flooded rice fields. This aligns with our observations and previous findings that rice farming did not have a large gender disparity^30^. We found that even in this population of farmers, people spent a large proportion of their daytime hours in the village, though this pattern was gendered and corresponded to seasonal farming activities (e.g., vanilla curing occurs in the village), the presence of young children in the household, and larger households. The gendered response to time spent outside the village is noteworthy because it provides a mechanism for gender-based differences in disease exposure. For example, women have been shown to have a higher prevalence of smoke inhalation-related disease than men because of cooking, an activity that primarily occurs in the village^31^. The complementary response—men spending more time outside of the village—exposes men to farming-related injuries and soil-transmitted helminths that cannot survive the sun-exposed soils in the village^32,33^. GPS trackers are particularly useful for obtaining data on environmentally transmitted diseases, because they estimate time in areas likely to be reported in a survey (i.e., crop fields) as well as places likely to be missed (i.e., paths and brief excursions).

Sociodemographic traits also explain variations in land use and home range size, albeit the effect size of many of these indicators was small. More educational attainment corresponded to smaller home range size and more time spent in secondary forests. This association may reflect long-term familial wealth (e.g., parents paid for schooling) or more time cultivating vanilla. Education may indicate a household life cycle pattern; however, we collected individual-level, not household-level data, and thus were limited in assessing this previously observed pattern^34^. Older individuals, despite expectations of mobility limitations, had larger home range sizes and spent more time outside the village. This is consistent with an enhanced livelihood diversity portfolio strategy^35^ as individuals with greater long-term wealth spent less time in brushy regrowth and flooded rice fields, farmed more types of crops—especially cash crops—and owned more animals. In contrast, individuals with less house-based or goods-based wealth and greater crop and animal diversity, yet lower-value animals, spent more time in brushy and bare areas where swidden agriculture occurs. This is consistent with a resource-poor risk-aversion strategy^36^.

The relationship between age, wealth, and land use reveals potential intervention points for easing acute economic needs within local farming communities and enhancing global conservation and infectious disease prevention efforts^3,37^. Understanding the sociodemographic correlates of forest use within Marojejy National Park is important to individual exposure to forest-associated infectious diseases and local conservation activities, as it likely reflects illegal harvesting of wood^32,38^. For example, younger men with young children who mostly farm subsistence crops and own fewer animals are more likely to enter the semi-intact forest inside the national park. This is consistent with younger individuals who have greater capability for strenuous tasks such as logging and our observations and casual conversations with community members about why individuals enter the national park. The park-use data also pinpoints a potential conservation lever: providing short term financial assistance to families that have recently invested in agriculture may prevent near-term illegal logging while families await a return on their investment. Also, notable is that park use is more likely to occur in the villages (B and C) where the economic benefits of national park tourism are lower and are less likely to occur during the transitional season (season 3). Another potential conservation intervention point (e.g., government policy, non-government initiatives) revealed through the GPS and survey data is the need for education on sustainable agricultural techniques, particularly among individuals who primarily farm subsistence crops. This could improve local crop yields, thus protecting food security and livelihood^25^. In the long term, more sustainable agricultural practices, particularly those that stabilize hillside soil horizons, would benefit the communities by preventing soil runoff into flooded rice fields, which lowers yields in these high-productivity areas^3,25,32^.

Previous findings on wealth and land use in LMICs found that wealth indices are often uncorrelated and fail to capture the multidimensional aspects of poverty^39^. Disentangling long-term social standing and stability (wealth) from short-term circumstances and challenges (income) using data obtained in a survey is complex, and different methods of calculating wealth indices might have yielded different results. Our work highlights that in studies of land use patterns among farmers in LMICs, income should be considered alongside long-term wealth indicators because it drives and reflects investments in daily activities. This is particularly important when considering internal deforestation by individuals living in areas where fertile land availability is limited and investment in capital-intensive crops is increasing^40,41^.

Deriving movement-based land use patterns using remotely sensed data and using GPS loggers have several limitations. First, presence in a particular land cover type does not provide information about the activities that individuals carry out in that place. Similarly, land cover classifications provide broad categories of spectrally similar areas, not the specific crops grown in any location, thus our inference is constrained to assumptions about typical activities in a given land cover type. Furthermore, GPS logger data is subject to participants’ willingness to wear the logger consistently and can only provide insights to land use during time the device was worn. These limitations provide alternate interpretations of the land use patterns. For instance, the rice farmers in our study spent relatively little time in areas classified as flooded rice compared to other land cover types. This may be an image classification issue due to hillside rice fields being classified as brushy vegetation or bare ground, due to difficulty distinguishing among classes between cropping periods. Alternatively, individuals may have removed the GPS device while working in a flooded field to prevent it from getting wet or interfering with their work. Likewise, people may not have worn their GPS tracker inside the national park due to legal concerns, which limit the conclusions we can draw about that cover type. While we were able to remove complete days a person did not wear a tracker, we cannot confidently remove shorter non-wear periods (discussed in Kauffman et al.^27^). However, our observations of land use and experience working in these villages, along with rigorous quality control steps, reduced, if not eliminated, these biases. The conclusions drawn from GPS tracking data are limited to the period that people wore the device, however, the study spanned multiple season and villages so collectively the dataset spans multiple years. Our model accounts for both the length of time individuals’ wear a GPS and the village and season in which the data were collected thus allowing for a broad understanding of sociodemographics and land use.

Movement ecology approaches provide a richer understanding of the demographic predictors of human land use while avoiding self-reporting biases^7,10^ and incorporating landscape scale land cover and use information. Studying who uses what types of land is important for understanding environmentally mediated disease exposure and protecting forest frontiers, though private GPS data should not be used in policing and instead anonymized formats are useful in developing targeted policy and NGO-based interventions. Disparities in land use and disease are central to the health of farmers in LMICs living in areas undergoing rapid environmental shifts due to a changing climate. Our findings reveal strong connections between sociodemographic variables and individual land use, which are important for developing sustainable solutions to limit deforestation, protect individual health, and advance adaptive farming practices to increase food security and livelihood resilience^3,42^. This research can inform theory and policy interventions in other locations globally where human mobility on the agricultural frontier is intimately connected to health, livelihoods, and environmental conservation.

## Methods

### Study location and design

The study took place from 2019 to 2022 in three communities (herein “villages”) along the boundary of Marojejy National Park in the SAVA region of northeastern Madagascar. Villages were chosen based on their proximity to the national park, size, accessibility, and agricultural activities. Village A has been the subject of much research by our group for many years, thus villages B and C were selected because they are similar to village A in these characteristics. Each village was surveyed over 3 “seasons”: season 1 is hot and dry, 2 is warm and wet, and 3 is wet and cool (transitional), for 3–6 weeks each season (Supplementary Table 4). Village A is the tourist entrance to Marojejy National Park. Village B is located near the city of Andapa (Fig. 1). Village C was the most remote and consisted of two closely connected communities (< 1 km apart) with strong familial ties and shared use of farmland, and thus we considered them a single village. The estimated population sizes of the villages were 2700, 900, and 1900, respectively. The area surrounding village A is a broad, large valley with relatively little elevational variation and many streams. It neighbors a river, the edges of which are largely wooded with natural secondary vegetational cover. Village B is situated along a river within a fertile basin at the base of a steep incline into the National Park. Village C is situated along a steep valley with small streams and features a river and little native vegetation.

Participants (≥ 18 years) were enrolled in the study using snowball sampling^43^, starting with a small group of landowners and their households. Other individuals in the community became eligible for enrollment when they were named as someone with whom enrolled individuals spend their free time, exchange farmwork, or exchange food. A large proportion of these tight knit communities were sampled and we expect had sampling continued their entire adult population would have been sampled, thus minimizing bias of the enrollment method.

### Inclusion and ethics statement

Study protocols were approved by the Duke University Institutional Review Board (Duke IRB Number 2019–0560) and the Ethics Committee for Biomedical Research within the Ministry of Public Health in Madagascar (Permit Number 114 MSANP/SG/AGMED/CERBM). All research was performed in accordance with relevant regulations, and informed consent was obtained from all study participants. Participants gave oral consent for the survey and written consent for the GPS trackers, which included an information sheet on the technology and how the data would be secured and used.

Local researchers led the data collection effort. Our Malagasy-speaking coauthors worked with English-speaking social scientists to translate the study questions to ensure the intent and meaning of the questions were correct. The survey was pilot-tested with a small group of community members in the villages to ensure clarity and that the questions were capturing the information our study aimed to collect given local economic nuances. All aspects of the study were explained by trained Malagasy assistants who were also accompanied by a person well known in the village and to whom participants would feel comfortable asking questions or declining to participate. We also held community meetings and consulted with village leaders at the start end and end of each survey mission to brief people on how and why we were conducting the survey. At the end of our study results were shared with relevant local, regional, and national authorities, who then reported back to the communities in which we worked.

### Sociodemographic survey

We collected survey data on demographics and wealth. Surveys were administered in the local dialect by trained Malagasy researchers from the same region. Responses were recorded on tablets using Qualtrics survey software (Qualtrics, Provo, Utah). Participants reported their age, gender, household composition, educational attainment, marital status, primary occupation, the amount of land owned, and information on house construction materials, durable goods owned, number of domestic animals owned, and types of crops grown. We created wealth indices based on household construction materials, ownership of durable goods, crops grown, and animal ownership. See below for detailed methods of the wealth indicator PCAs.

After completing the survey, participants could choose to wear a GPS tracker for 1 week, up to the remaining number of weeks in the study period. We explained to participants how the trackers worked, including showing them a map of the area with the trajectories of ourselves and/or domestic animals plotted and language that stated “people’s movements will be tracked” by these devices. GPS data loggers (iGot-U 120; Mobile Action, New Taipei City, Taiwan) recorded GPS location every 3 min. Freshly charged devices were distributed weekly for up to 6 weeks.

A small compensation for participating in the survey and wearing GPS trackers was given in the form of pre-paid phone credits: 1000Ar survey, 3000Ar per week the device was worn. We did not require participants to finish the survey, nor did we quality check GPS tracks as a requisite for compensation.

### Land cover classification

Cloud-free satellite imagery of the study region was averaged across three field seasons and used to build a land cover classification model in ArcGIS. Training data consisted of ground-truthed polygons from each village and polygons drawn manually using high-resolution imagery. The classified images were then manually edited to ensure that areas with high human use were classified correctly. The full methods used to prepare the image classification schema are detailed in Kauffman et al.^27^. Table 2 provides details on the land-use types encompassed in each class and activities that typically occur in these land cover types based on our knowledge of the area.

### GPS-based movement analysis

The GPS-based tracking data were processed as described in Kauffman et al.^27^ and summarized in Supplementary Table 5. Briefly, we first removed erroneous points, the distribution day, and days that participants did not wear the device, determined by self-reporting and individuals’ daily minimum convex polygon and trajectory. Next, the study area around each village was determined using a minimum convex polygon based on all the locations of all individuals while excluding excursions away from the local area. Both visualization of the trajectories and an elbow plot of study area size were used to minimize area while maximizing data inclusion. We removed excursions because we wanted to understand land use within the vicinity of the village and because people generally returned their GPS before traveling. Furthermore, removing excursions limited the impact the COVID-19 pandemic may have had on land use patterns since travel, not routine farming activities around home, were the most likely impacted behavior. Individuals’ daytime (06:00 to 19:59 GMT + 3) utilization distributions (UD), which are the probability density estimates of individuals occurring at a given location^17^, were calculated using a dynamic Brownian bridge movement model^29^. UDs are useful for understanding the extent of areas used and correlating occurrence probability with underlying environmental features^18^. Analysis was limited to daytime hours to capture the active period, rather than when people were stationary at home or asleep.

The size of an individual’s home range (number of pixels) and their intensity of land use (mean pixel value of time spent within the home range) were found using the *global* function in the *terra* package^44^. The zonal function was used to calculate the total time spent (proportion of their home range) and area used (number of pixels within their home range) within each cover type.

### Statistical analysis

All analyses were conducted in R version 4.4.1^45^.

### Wealth indicators

Wealth indicators for house construction materials, durable goods owned, crops farmed, and animals owned were built following the DHS-8 guidelines^46^ to use principal component analysis (PCA) and recommendations made by Poirier et al.^39^. We also considered the amount of land a participant owned as a single-dimensional indicator of wealth. Using multiple indicators of types of wealth is well suited to rural, LMIC settings, and aligns with our aim of obtaining a more comprehensive understanding of how socioeconomics influence land use instead of the influence of single attributes (e.g., animal or crop types).

To transform the categorical responses on house construction materials into a numeric response to use in the PCA we ranked the primary construction material used for participants’ walls, floor, and roof from one to four starting with gathered natural materials, then improved materials (wood planks, bricks), purchased metal sheeting, and purchased cement. Also within the house construction materials was the type of latrine owned, which was similarly ranked from no latrine (1), pit latrine (2), to improved pit latrine (4). The greater ranking difference for an improved pit latrine was due to the substantial increase in value which is on par with that of using cement in house construction materials. Ownership of nine durable goods was scored—car, refrigerator, computer, generator, motorcycle, bicycle, television, radio, cell phone and as a 10th category to capture market integration and wealth, we asked whether the person used phone banking. Participants reported all the crops they grew. For this analysis, we limited crops to those that at least 50 people reported growing (vanilla, rice, cassava, banana, coffee, sugar cane, cloves, maize, beans, nuts, taro, onion, pineapple, and coconut). The crops omitted were primarily vegetables and fruit trees which people grew for their own consumption.

Participants also reported the number of cats, dogs, chickens, ducks, geese, turkeys, cows, goats, pigs, sheep, rabbits, and fish they owned. The number of each was recorded as 1 to more than 5, which was coded as 6. As poultry type was not considered important for assessing wealth or income, we summed together all poultry (chickens, ducks, geese, turkeys) and then scaled this (divided by 4) to match the other animals.

We used the *rda* function in the *vegan* package to conduct the PCA^47^ for each multivariate wealth indicator. We then found Pearson’s correlation between indicators to assess if they were capturing similar aspects of wealth. We also checked if including all indicators in the generalized linear models caused multicollinearity using the *car* package^48^.

### Generalized linear models

We built two sets of generalized linear models on the size of an individual’s home range (ha, log_10_) and the amount of time (proportion of home range) individuals spent in different land classifications. Covariates in these models were village, study season, gender, age, school level, marital status, amount of land owned, household size, whether the household had any young children (under 3 years of age), the interaction between gender and having young children, and wealth.

We built the generalized linear models using the *glmmTMB* package^49^, assessed residuals using the *DHARMa* package^50^, and assessed model fit using the corrected Akaike Information Criterion corrected for small sample sizes (AICc). We used weighted models to account for variation in the amount of time an individual wore a GPS data logger, with weights calculated as 1 + log(number of days GPS logger was worn). Individuals that wore a GPS for longer, were likely to have data that was more representative of their land use patterns (i.e., a person visits a greater extent of the area they typically use, given more time). Response variables were transformed as needed to ensure model fit: home range size was log_10_ transformed, time in the village was changed to time outside of the village to match the right-skewed distributions of the other land-classification types, and we used a binomial response for time in the semi-intact forest instead of the proportion of time spent there due to sparsity. We used the dredge function in the *MuMIN* package^28^ to identify the best subset of models (∆AICc ≤ 2), assess the importance of individual predictors (sum AICc weight [sw] for all models containing the covariate), and find the full model averaged effects. Tukey-adjusted post-hoc comparisons were made using a model containing only important (sw = 1) covariates for the respective response using the *emmeans* package^51^.

## Supplementary Information

Below is the link to the electronic supplementary material.

Supplementary Material 1

## Data Availability

The survey and GPS datasets generated during and analyzed during the current study are not publicly available due to privacy or ethical restrictions but are available from the corresponding author on reasonable request. The classified land cover file, the code and a pdf document of the data analysis is available at https://osf.io/zfsy3/.
