# Supplementary material for: Sociodemographic characteristics predict land use patterns by farmers near a protected area in Madagascar
Source: Sci Rep. 2026 Feb 22;16:9193. doi: 10.1038/s41598-026-40592-6 (PMC12996304; doi:10.1038/s41598-026-40592-6)
Supplement: Supplementary file 1 — Supplementary Material 1 [file 41598_2026_40592_MOESM1_ESM.docx]

# Sociodemographic characteristics predict land use patterns by farmers near a protected area in Madagascar

Contents

[Sociodemographic characteristics predict land use patterns by farmers near a protected area in Madagascar 1](#_Toc221088570)

[Supplementary Tables 3](#_Toc221088571)

[Supplementary Table 1. The percentage and area (km2) of each land cover classification in the study area around each village. 3](#_Toc221088572)

[**Supplementary Table 2**. The importance of each fixed effect is based on the sum weight of each model containing the predictor in the “best model subset” by ∆AICc ≤2. The number of models in the best model subset is indicated in parentheses. Each cell's shade indicates the predictor's importance for that model. The response variable in the home range model is the home range size in hectares, in the proportion of home range models (Bare, Brushy, Rice, Secondary, Outside Village) is the proportion of the home range spent in the indicated land cover classification type. 4](#_Toc221088573)

[**Supplementary Table 3.** Full model averaged coefficients and 95% confidence intervals. 5](#_Toc221088574)

[**Supplementary Table 4.** Survey dates by village and season. 6](#_Toc221088575)

[**Supplementary Table 5.** The percentage of GPS points sequentially removed based on the quality filter in each column from left to right. 6](#_Toc221088576)

[Supplementary Figures 7](#_Toc221088577)

[**Supplementary Figure 1**. Wealth and socio-economic indicators biplots of the first two principal components (PC) generated from principal component analysis using the *rda* function in the vegan package ^1^ for responses in each category: (A) house construction material ranked values, (B) binary ownership of durable goods, (C) binary crops grown for all crops that >50 survey respondents grew, and (D) then number of each type of animal owned. The points represent the individual responses, the vectors indicate the amount of variation in responses explained by the corresponding variable along PC1 and PC2, and the dashed lines represent 95% confidence convex hulls for each village. The percentage on each axis indicated the proportion of variation in the dataset explained by the respective component. Figures were made using the ggplot2 ^2^ and patchwork ^3^ packages. 7](#_Toc221088578)

[**Supplementary Figure 2. The proportion of cover type available and used by village.** The available category means the proportion of each cover type within the study area around each village, and accessibility of all parts of the study area was not assessed. The used category means that ≥1 individual’s home range included that part of the study area. 8](#_Toc221088579)

[References 9](#_Toc221088580)

## Supplementary Tables

| village | Semi-intact | Secondary | Rice | Village | Brushy | Water | Bare | Total |
| --- | --- | --- | --- | --- | --- | --- | --- | --- |
| A | 3.87% (1.92) | 46.47% (23.07) | 6.75% (3.35) | 1.73% (0.86) | 35.61% (17.68) | 2.23% (1.11) | 3.35% (1.67) | 100% (49.67) |
| B | 6.22% (2.17) | 13.34% (4.66) | 31.37% (10.96) | 2.76% (0.96) | 21.68% (7.57) | 1.36% (0.47) | 23.26% (8.12) | 100 (34.93) |
| C | 9.58% (1.93) | 36.41% (7.34) | 8.89% (1.79) | 1.08% (0.22) | 38.95% (7.85) | 0.98% (0.20) | 4.11% (0.83) | 100% (20.16) |

### Supplementary Table 1. The percentage and area (km2) of each land cover classification in the study area around each village.

|  | Home- range | Bare | Brushy | Rice | Secondary | Semi intact | Outside Village |  |  |
| --- | --- | --- | --- | --- | --- | --- | --- | --- | --- |
| Study village | 1 (12) | 1 (12) | 1 (6) | 1 (15) | 1 (6) | 1 (10) | 1 (5) |  |  |
| Study season | 1 (12) | 1 (12) | 1 (6) | 0.48 (7) | 1 (6) | 1 (10) | 1 (5) |  |  |
| Gender | 1 (12) | 1 (12) | 1 (6) | 1 (15) | 1 (6) | 1 (10) | 1 (5) |  | Importance |
| Age | 0.06 (1) | 1 (12) | 1 (6) |  | 1 (6) | 1 (10) | 1 (5) |  | 1 |
| Schooling | 1 (12) | 0.14 (2) |  | 0.83 (12) | 1 (6) |  |  |  | 0.8-0.99 |
| Marital status | 0.21 (3) | 0.59 (7) | 0.11 (1) |  | 0.88 (5) | 0.16 (2) |  |  | 0.6-0.79 |
| Land size | 0.13 (2) | 0.86 (10) | 1 (6) | 0.67 (10) | 0.86 (5) |  | 1 (5) |  | 0.4-0.59 |
| Household size |  | 1 (12) | 0.64 (4) |  |  | 0.40 (4) | 1 (5) |  | 0.2-0.39 |
| Children under 3 | 0.84 (10) | 1 (12) | 0.26 (2) | 0.08 (2) | 1 (6) | 1 (10) | 0.11 (1) |  | 0.01-0.19 |
| Gender*Children | 0.33 (4) | 1 (12) |  |  | 0.11 (1) | 1 (10) |  |  | 0 |
| House construction PC1 | 0.06 (1) | 0.21 (3) | 1 (6) |  | 0.11 (1) | 0.07 (1) | 0.31 (2) |  |  |
| Durable goods PC1 | 1 (12) | 0.76 (9) |  | 1 (15) | 1 (6) | 0.08 (1) |  |  |  |
| Crops PC1 | 1 (12) | 0.05 (1) | 1 (6) | 0.75 (11) | 1 (6) | 0.08 (1) | 1 (5) |  |  |
| Crops PC2 | 0.84 (10) | 1 (12) | 0.13 (1) |  | 0.23 (1) | 1 (10) | 0.41 (2) |  |  |
| Animals PC1 | 1 (12) | 1 (12) | 1 (6) | 1 (15) | 1 (6) | 0.91 (9) | 1 (5) |  |  |
| Animals PC2 |  | 1 (12) | 1 (6) | 0.89 (13) | 1 (6) | 0.21 (2) | 1 (5) |  |  |

### **Supplementary Table 2**. The importance of each fixed effect is based on the sum weight of each model containing the predictor in the “best model subset” by ∆AICc ≤2. The number of models in the best model subset is indicated in parentheses. Each cell's shade indicates the predictor's importance for that model. The response variable in the home range model is the home range size in hectares, in the proportion of home range models (Bare, Brushy, Rice, Secondary, Outside Village) is the proportion of the home range spent in the indicated land cover classification type.

|  | Home range | Bare | Brushy | Rice | Secondary | Semi intact | Outside Village |
| --- | --- | --- | --- | --- | --- | --- | --- |
| Intercept | 0.5626 [0.2021,1.0312] | -0.9884 [-0.9913,-0.9845] | -0.908 [-0.9221,-0.8914] | -0.9301 [-0.9438,-0.9129] | -0.927 [-0.9451,-0.903] | -0.9725 [-0.9888,-0.9324] | -0.6557 [-0.7113,-0.5894] |
| Zero-inflation intercept | NA | NA | -0.9797 [-0.985,-0.9725] | -0.942 [-0.9518,-0.9301] | NA | NA | -0.9891 [-0.9928,-0.9836] |
| village: B | -0.2583 [-0.3545,-0.1478] | 8.1265 [6.8229,9.6473] | -0.2584 [-0.3343,-0.1739] | -0.2645 [-0.3488,-0.1694] | -0.6947 [-0.731,-0.6533] | 2.5742 [1.0086,5.3602] | -0.2072 [-0.2961,-0.1071] |
| village: C | -0.1617 [-0.259,-0.0517] | 0.3962 [0.2072,0.6148] | 0.1334 [0.0338,0.2425] | -0.0607 [-0.1559,0.0454] | -0.0187 [-0.1145,0.0875] | 1.4075 [0.3905,3.1684] | 0.0333 [-0.0691,0.1469] |
| season: 2 | -0.187 [-0.2707,-0.0938] | -0.15 [-0.2438,-0.0446] | 0.0379 [-0.0457,0.1288] | 0.0084 [-0.0588,0.0805] | 0.2474 [0.1366,0.3691] | -0.3232 [-0.543,0.0025] | 0.0918 [-0.0056,0.1987] |
| season: 3 | -0.2689 [-0.36,-0.1648] | -0.4273 [-0.5016,-0.3419] | -0.2886 [-0.3611,-0.2078] | 0.0577 [-0.0816,0.2182] | -0.3819 [-0.4538,-0.3006] | -0.5818 [-0.7451,-0.3138] | -0.2583 [-0.3398,-0.1668] |
| gender: Male | 0.8499 [0.6607,1.0606] | 0.1252 [5e-04,0.2655] | 0.2575 [0.1688,0.3529] | 0.1034 [0.0134,0.2013] | 0.2333 [0.1333,0.342] | 3.052 [1.3542,5.9744] | 0.3036 [0.204,0.4115] |
| Age (decades) | 5e-04 [-0.0082,0.0092] | 0.0487 [0.0128,0.086] | 0.0335 [0.0099,0.0577] | NA | 0.0923 [0.0614,0.124] | -0.1131 [-0.2105,-0.0038] | 0.0685 [0.0415,0.0962] |
| school: 1° | -0.1989 [-0.3781,0.0318] | 0.0328 [-0.144,0.2461] | NA | 0.0827 [-0.1246,0.3392] | 0.0715 [-0.1431,0.3399] | NA | NA |
| school: 2° | -0.1806 [-0.3664,0.0597] | 0.0289 [-0.1356,0.2247] | NA | 0.0404 [-0.1542,0.2796] | 0.1843 [-0.0594,0.4913] | NA | NA |
| school: 3° | -0.3716 [-0.5255,-0.1679] | 0.013 [-0.1117,0.1552] | NA | -0.0696 [-0.2633,0.175] | 0.3029 [0.011,0.6792] | NA | NA |
| Marital Status: Partner | 0.0121 [-0.0564,0.0855] | 0.0621 [-0.0775,0.2229] | 0.0021 [-0.029,0.0341] | NA | -0.0865 [-0.1851,0.024] | 0.0281 [-0.1725,0.2773] | NA |
| Landsize log_10_ hectares | -2e-04 [-0.0022,0.0018] | -0.004 [-0.0096,0.0015] | -0.0048 [-0.0086,-0.001] | -0.0023 [-0.0069,0.0024] | -0.0037 [-0.0087,0.0014] | NA | -0.0069 [-0.011,-0.0029] |
| household size | NA | -0.061 [-0.089,-0.0322] | -0.01 [-0.0311,0.0116] | NA | NA | -0.0282 [-0.1163,0.0688] | -0.023 [-0.0438,-0.0018] |
| child: yes | -0.0913 [-0.2188,0.0571] | -0.1245 [-0.2551,0.0289] | -0.0093 [-0.0592,0.0431] | 0.0013 [-0.0248,0.0282] | -0.1125 [-0.1922,-0.0249] | -0.6501 [-0.8965,0.1832] | 0.0015 [-0.0291,0.0331] |
| gender*child | 0.0439 [-0.1131,0.2287] | 0.3201 [0.0799,0.6137] | NA | NA | 0.0033 [-0.0543,0.0645] | 2.9923 [0.1263,13.151] | NA |
| house construction PC1 | -9e-04 [-0.0145,0.013] | 0.0054 [-0.0262,0.038] | -0.0442 [-0.0794,-0.0078] | NA | -0.0012 [-0.0171,0.015] | -0.0036 [-0.0565,0.0523] | -0.0065 [-0.0356,0.0234] |
| durable goods PC1 | 0.0659 [0.0161,0.1183] | 0.0365 [-0.0245,0.1014] | NA | -0.047 [-0.0872,-0.0051] | 0.0572 [0.0117,0.1046] | -0.004 [-0.0567,0.0516] | NA |
| crops PC1 | 0.0747 [0.0253,0.1265] | -5e-04 [-0.0126,0.0117] | 0.079 [0.0393,0.1203] | 0.029 [-0.0199,0.0803] | 0.0457 [0.0032,0.0899] | 0.004 [-0.0473,0.0581] | 0.0728 [0.0304,0.1169] |
| crops PC2 | -0.034 [-0.0822,0.0167] | 0.074 [0.0251,0.1253] | -0.0019 [-0.018,0.0144] | NA | -0.0059 [-0.0343,0.0232] | 0.2562 [0.0734,0.4703] | -0.0107 [-0.0451,0.025] |
| animals PC1 | 0.0844 [0.0367,0.1343] | 0.1438 [0.089,0.2014] | 0.0731 [0.0349,0.1127] | 0.1767 [0.1331,0.2219] | 0.0603 [0.0202,0.1019] | -0.1643 [-0.3277,0.0387] | 0.1586 [0.1142,0.2048] |
| animals PC2 | NA | 0.1018 [0.0501,0.156] | 0.0596 [0.0241,0.0964] | -0.0303 [-0.0689,0.0099] | 0.1512 [0.1079,0.1963] | 0.0215 [-0.0882,0.1443] | 0.0593 [0.0199,0.1002] |

### **Supplementary Table 3.** Full model averaged coefficients and 95% confidence intervals.

| Village | Season 1 | Season 2 | Season 3 |
| --- | --- | --- | --- |
| A | 3 Oct - 9 Nov 2019 (6 weeks) | 2 March - 16 March 2020 (2 weeks)* | * |
| B | 9 December - 26 December 2020 (3 weeks)* | 21 March - 6 May 2021 (6 weeks) | 26 July - 9 August 2021 (6 weeks) |
| C | 25 October - 9 December 2021 (6 weeks) | 8 March - 21 April 2022 (6 weeks) | 21 June - 4 August (6 weeks) |

### **Supplementary Table 4.** Survey dates by village and season.

*survey was cut short or omitted due to COVID-19 pandemic

| Village | Erroneous | GPS distribution day | GPS not worn | Outside study area | Points used in analysis |
| --- | --- | --- | --- | --- | --- |
| A | 0.25% (1912/769890) | 1.17% (9014/767978) | 25.63% (194542/758964) | 6.23% (35186/564422) | 68.74% (529236/769890) |
| B | 0.38% (4836/1277092) | 1.5% (19021/1272256) | 56.91% (713268/1253235) | 3.04% (16398/539967) | 41% (523569/1277092) |
| C | 0.34% (5421/1598254) | 3.09% (49235/1592833) | 53.9% (802875/1489572) | 6.54% (48052/734749) | 42.97% (686697/1598254) |

### **Supplementary Table 5.** The percentage of GPS points sequentially removed based on the quality filter in each column from left to right.

## Supplementary Figures

| 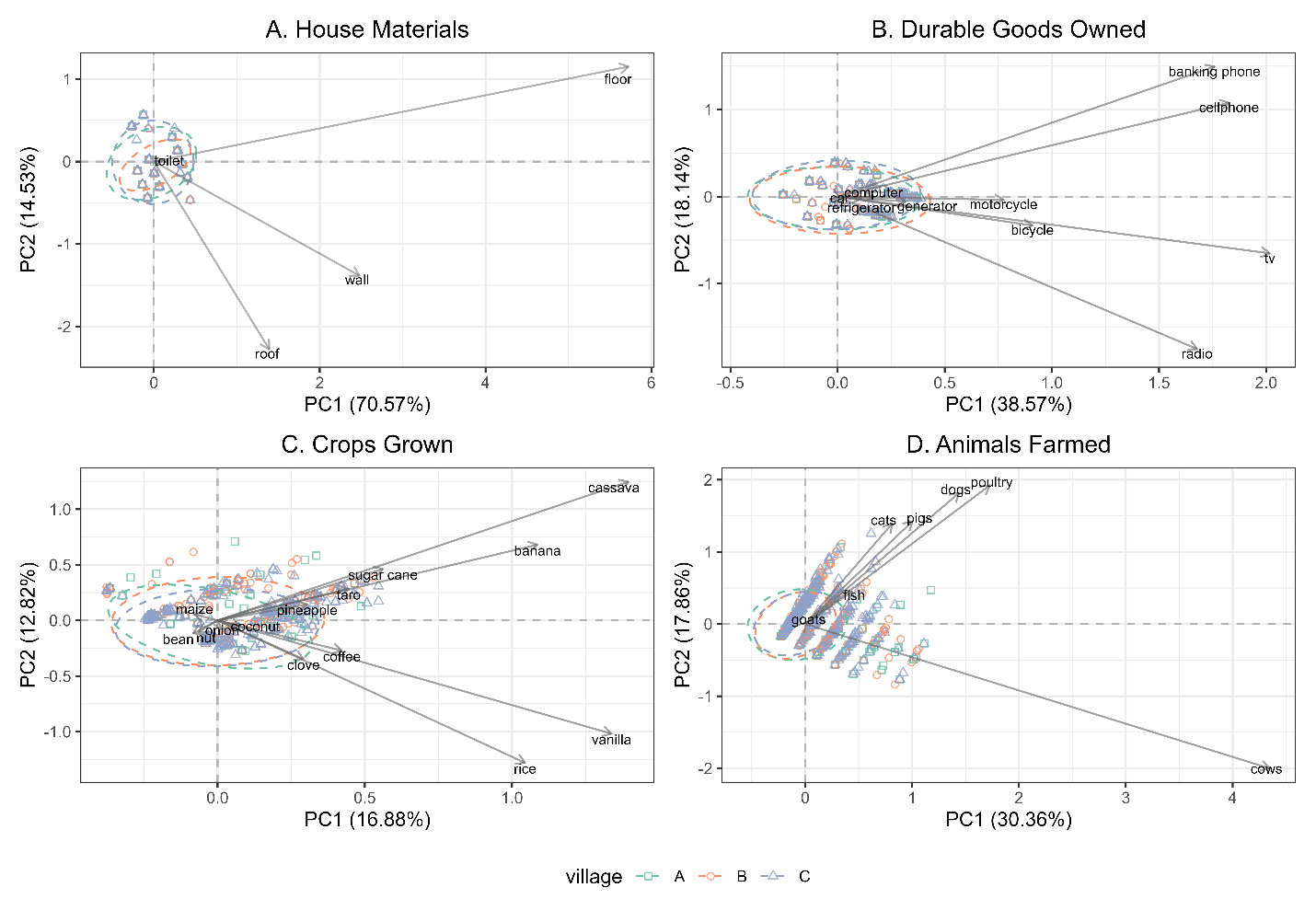 |
| --- |
| **Supplementary Figure 1**. Wealth and socio-economic indicators biplots of the first two principal components (PC) generated from principal component analysis using the *rda* function in the vegan package ^1^ for responses in each category: (A) house construction material ranked values, (B) binary ownership of durable goods, (C) binary crops grown for all crops that >50 survey respondents grew, and (D) then number of each type of animal owned. The points represent the individual responses, the vectors indicate the amount of variation in responses explained by the corresponding variable along PC1 and PC2, and the dashed lines represent 95% confidence convex hulls for each village. The percentage on each axis indicated the proportion of variation in the dataset explained by the respective component. Figures were made using the ggplot2 ^2^ and patchwork ^3^ packages. |
| **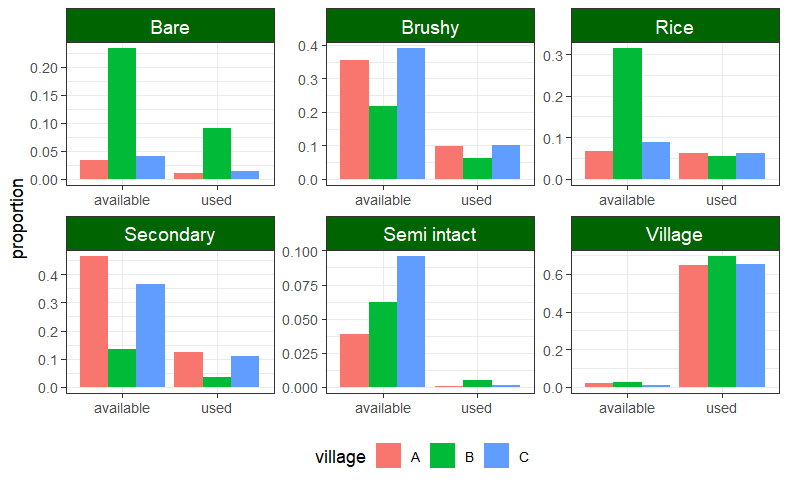** |
| **Supplementary Figure 2. The proportion of cover type available and used by village.** The available category means the proportion of each cover type within the study area around each village, and accessibility of all parts of the study area was not assessed. The used category means that ≥1 individual’s home range included that part of the study area. |

# References

1. Oksanen, J. *et al.* *Vegan: Community Ecology Package*. (2024).

2. Wickham, H. *Ggplot2: Elegant Graphics for Data Analysis*. (Springer-Verlag New York, 2016).

3. Pedersen, T. L. *Patchwork: The Composer of Plots*. (2024).
